# Current Treatment Strategies for Intracranial Aneurysms: An Overview

**DOI:** 10.1177/0003319717700503

**Published:** 2017-03-30

**Authors:** Junjie Zhao, Hao Lin, Richard Summers, Mingmin Yang, Brian G. Cousins, Janice Tsui

**Affiliations:** 1 Division of Surgery & Interventional Science, UCL Centre for Nanotechnology and Regenerative Medicine, University College London, London, United Kingdom; 2 Guangdong Provincial Hospital of TCM, Guangzhou, People’s Republic of China; 3 Swansea University, Wales, United Kingdom; 4 Department of Cell Biology, UCL Institute of Ophthalmology, University College London, London, United Kingdom; 5 Royal Free London NHS Foundation Trust Hospital, London, United Kingdom; * Authors equally contributed to this manuscript

**Keywords:** stroke, intracranial aneurysm, angiography, cerebral revascularization, stenting, intracranial vasospasm

## Abstract

Intracranial aneurysm is a leading cause of stroke. Its treatment has evolved over the past 2 decades. This review summarizes the treatment strategies for intracranial aneurysms from 3 different perspectives: open surgery approach, transluminal treatment approach, and new technologies being used or trialed. We introduce most of the available treatment techniques in detail, including contralateral clipping, wrapping and clipping, double catheters assisting coiling and waffle-cone technique, and so on. Data from major trials such as Analysis of Treatment by Endovascular approach of Non-ruptured Aneurysms (ATENA), Internal Subarachnoid Trial (ISAT), Clinical and Anatomical Results in the Treatment of Ruptured Intracranial Aneurysms (CLARITY), and Barrow Ruptured Aneurysm Trial (BRAT) as well as information from other clinical reports and local experience are reviewed to suggest a clinical pathway for treating different types of intracranial aneurysms. It will be a valuable supplement to the current existing guidelines. We hope it could help assisting real-time decision-making in clinical practices and also encourage advancements in managing the disease.

## Introduction

Intracranial aneurysms (IAs) are localized dilations of the cerebral arteries wall and are prone to rupture, resulting in bleeding. The overall prevalence of unruptured IAs is between 2% and 3.2% in the general population with a male to female ratio of 1:2.^1^ It is the leading cause of hemorrhagic stroke, responsible for 85% of subarachnoid hemorrhages (SAH).^2^ In the United Kingdom, approximately 10% to 15% of patients with ruptured IAs die before reaching hospital. Of those who survive, 42% will be dependent, 46% will have some form of disability, and 12% will be left severely impaired.^3^ The treatment techniques and management guidelines for IAs have been continually developing since the 1990s. This rapid development has caused difficulty for clinicians in the field to respond to the changes and operate within the ever-changing techniques and guidelines. The purpose of this review is to provide young practitioners, particularly those in surgical training, with an overview of the techniques available so as to have a better understanding of the management for the disease. In this review, we summarize the different types of IAs and various treatment strategies available.

## Different Types of IAs

### The 4 Basic Types of IAs

**Figure 1.**
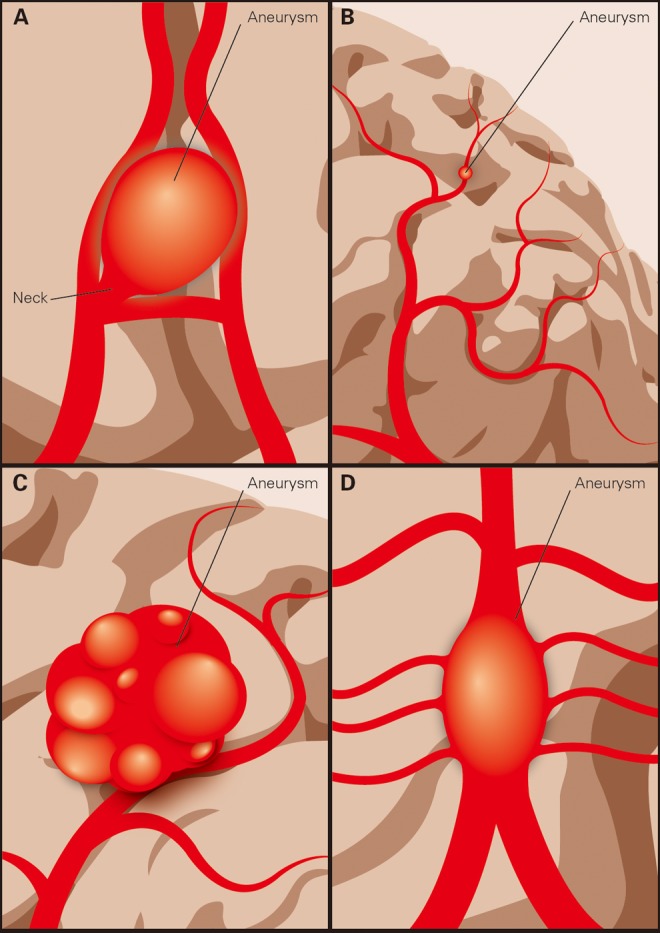
Types of IAs: (A) saccular IA, (B) microaneurysm, (C) GIA, and (D) fusiform IA. IAs indicates intracranial aneurysms; GIA, giant IAs.

Saccular IAs (Figure 1A) are the most common type of IAs. They resemble a round outpouching with well-defined aneurysmal domes and necks connecting to the parenting vessel. They favor bifurcation locations like between the middle cerebral artery (MCA) and the posterior cerebral artery (PCA), between the anterior cerebral artery (ACA) and the anterior cerebral artery (ACA) and the bifurcations of MCA branches.^4^

Microaneurysms (Figure 1B) are IAs with diameters smaller than 2 mm.^5^ Most microaneurysms are chronic hypertension related, also known as Charcot-Bouchard aneurysms. They often occur in blood vessels smaller than 0.3 mm and prone to the microvessels in the basal ganglia.^6^ The rest are infectious IAs (IIAs) or mycotic aneurysms, accounting for about 0.6% to 0.7% of all IAs.^7,8^ The IIAs in distal MCAs are related to septic emboli from infective endocarditis, while proximal branches are more likely to be affected via infection spread from cavernous sinus thrombophlebitis or meningitis.^9^ The IIAs are usually small blister like in shape, and patients mostly present with infectious symptoms. Despite its low incidence, the morbidity and mortality are up to 80% in cases of ruptured IIAs.^9^

Giant IAs (GIAs; Figure 1C) are IAs with diameters over 25 mm. They account for only 5% of all IAs, but their prognosis is dismal.^10,11^ Untreated GIAs have over 50% risk in rupturing and 88% to 100% in mortality at 2-year follow-up.^12,13^ Due to their volumetric characteristics, the mass effect alone can cause intracranial hypertension and neurological dysfunctions.

Fusiform IA (Figure 1D) refers to a widened and thinning segment of artery. According to Yahia et al, the dilatation must affect at least 270^o^ of the lumen’s circumference to be classified as fusiform.^14^ Their treatment strategy lies in recanalization and is challenging both endovascularly and surgically due to the presence of vital perforators located within the diseased segment.

### Special Types of IAs

#### Dissecting aneurysms

**Figure 2.**
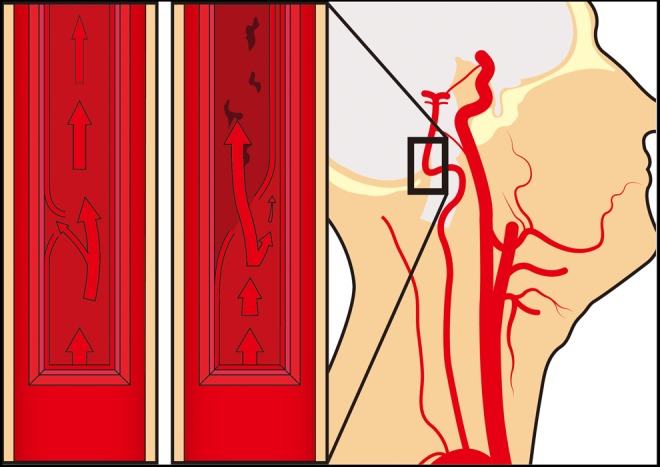
Schematic figure of dissecting aneurysm in VA. VA indicates vertebral artery.

Dissecting aneurysms, or arterial dissections, start with a minor tear on the inner wall, and then layers are further separated by the shearing force of blood flow which results in pseudoaneurysm formation. Most of them are trauma related as complications of endovascular intervention. Spontaneous dissections usually occur between V3 and V4 segments of the vertebral artery (VA; Figure 2). The curvy nature of VA promotes turbulence, leading to increased shear force. V3 navigates its path through ligaments with little mobility, and it breaks free from these bonds as it enters the dura. The turbulence contributes to free movements of V4, adding to the risk of tearing.^15^

#### Blood blister-like aneurysms

Blood blister-like aneurysms (BBAs) are defined as small aneurysms originating from nonbranching sites of the terminal internal carotid artery (ICA) with a broad base, which are assumed to be dissecting in nature.^16^ Despite its definition, they can also be found in other locations of the cerebral circulation. The BBAs are rare, accounting for 0.9% to 6.5% of all IAs.^17^ They have extremely fragile walls and are highly prone to spontaneous rupture. Little is known about their pathophysiology at present, but they are hypothesized to arise from dissections.^18^

#### Giant serpentine aneurysms

Giant serpentine aneurysm (GSA) is a subtype of GIAs. They were first described by Segal in 1977 as partially thrombosed aneurysms.^19^ The blood flowing through GSAs is slow, leading to repeated episodes of intraluminal clot formation. The clots build up and eventually block most of the aneurysmal lumen, leaving only a tortuous channel, which appears to be serpent-like under digital subtract angiograph (DSA).^20^ Due to their chronic nature, the thrombus inside is highly fibrosed, giving them stiff and rubber-like textures. The risks posed by GSAs are not bleeding but seizure or ischemic symptoms induced by their mass effects.^21^

#### “De novo” aneurysms

De novo aneurysms were first described by Graf and Hamby in 1964, referring to IA formation in previously normal locations that remote from the original lesion.^22^ They were rarely diagnosed due to the lack of follow-ups. The reported risk of de novo aneurysm formation was approximately 0.1% to 1.8%.^23^ The pathogenesis is most likely related to the hemodynamic changes induced by treatments.^24^ Zali et al suggested in their studies the risk of de novo formation was higher in patients with multiple IAs and higher in patients who had undergone surgical clipping than endovascular embolization.^23^

### Treatment Strategies for IAs

Conventional treatment options for IAs are either surgical or endovascular. However, conventional treatments are insufficient when dealing with special IAs or in complex cases. Innovations in both surgical and transluminal techniques have been developed over the past years, which have been summarized in the following sections.

## Surgical Techniques

### Simple Clipping

Simple surgical clipping refers to the practice of the exposure of the aneurysmal neck via craniotomy and the exclusion of the entire abnormal vascular wall from the circulation using single or multiple clips. Two principles apply in surgical clipping: isolating the lesion from active circulation and maintaining the integrity and patency of the parenting vessel. Simple clipping is suitable for most IAs, such as saccular IAs, GIAs, de novo IAs, and fusiform IAs without vital perforators branching from the lesion. The key of clipping surgeries lies in good neck exposure, and in cases where visual exposure and clip insertion is limited by the operating field, endoscope-assisted clipping can be used.^25^

### Contralateral MCA Aneurysm Clipping Technique

The contralateral MCA aneurysm clipping technique was first developed as an approach targeting IAs within a short distance from the midline, especially for those with domes branching out toward the midline.^26^ It is not preferable when dealing with common MCA aneurysms for the long dissection distance and difficulties presented in clip insertion. However, it presents advantages when dealing with bilateral MCA aneurysms. Bilateral MCA aneurysms account for 7.4% of MCA aneurysms and were treated with bilateral craniotomies in 2 separate stages. This technique spares the patients a second operation and is preferable as a less invasive approach.

### Temporary Artery Occlusion Technique

Aneurysmal rupturing is the primary concern during surgical clipping. Temporary artery occlusion (TAO) was first brought about in the 1960s by temporarily cutting off blood supply, the aneurysm shrinks, and allows the operator better vision and space to operate while preventing rupturing.^27^ Due to the potential for ischemic complications, the occlusion time applied is normally within 10 to 20 minutes, in complex cases, where a single TAO episode might not be sufficient, multiple episodes are applied with 15-minute reperfusions in between.^28^ It is still one of the most widely used techniques in clipping surgeries.

### Microscope-Integrated Near-Infrared Indocyanine Green Video Angiography

**Figure 3.**
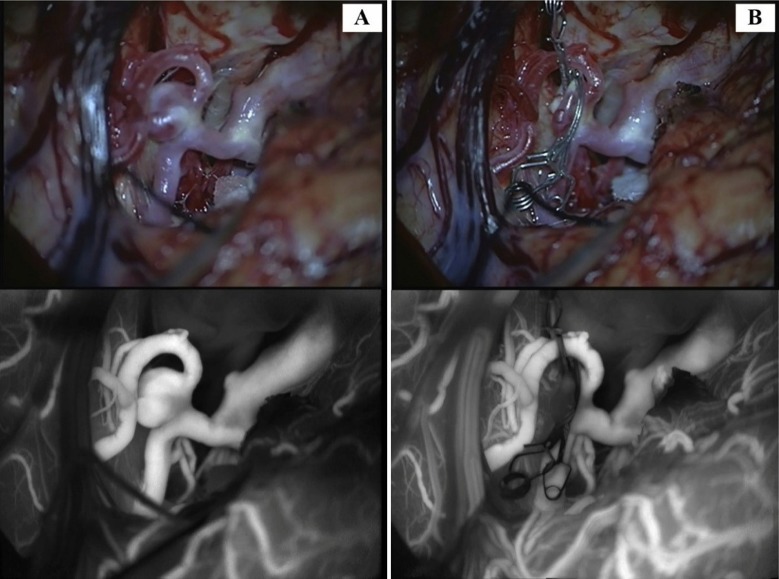
Intraoperation ICGVA images before and after clipping (upper: microscopic visualization; lower: infrared view of blood flow using ICGVA), A, MCA IA prior to clipping. B, MCA IA postclipping. ICGVA indicates microscope-integrated near-infrared indocyanine green video angiography; MCA, middle cerebral artery; IA, intracranial aneurysm.

Microscope-integrated near-infrared indocyanine green video angiography (ICGVA) was first applied by vascular neurosurgeons in 2005 to assess real-time intraoperative patency.^29^ Over the years, ICGVA has demonstrated efficacy and reliability in parenting vessel and perforator patency monitoring during surgery (Figure 3). A retrospective cohort by Lei et al suggested in 79% of the cases ICGVA was considered useful, and in 9% it was considered both crucial and critical in real-time decision-making.^30^

### Wrapping and Clipping

Wrapping and clipping is a technique applied to ruptured aneurysms where the lesion is wrapped with autogenous tissue or absorbable material to reconstruct the integrity of the vessel wall before clipping. This technique could be used for dealing with aneurysmal neck avulsions. Cotton fiber was used as wrapping material prior to the suggestion by Feng et al in 2013 that dura matter has higher efficiency in blocking perforation while maintaining patency.^31^ However, this method is not suitable for complete neck avulsion due to the deficit of aneurysmal neck or residual root; in such cases, an in situ bypass is required.^32,33^

Wrapping–clipping was once recommended for BBAs as simple clipping is hazardous for them and invariably results in aneurysmal avulsion with or without parent artery laceration.^17^ However, although data are lacking from large series clinical studies, it is described in many recent small studies and case reports that wrapping and clipping showed little improvement on the mortality or morbidity of BBAs in comparison to other surgical approaches such as simple clipping or ICA trapping with or without bypassing.^34,35^

### Bypass Techniques

#### Extracranial-to-intracranial bypass

**Figure 4.**
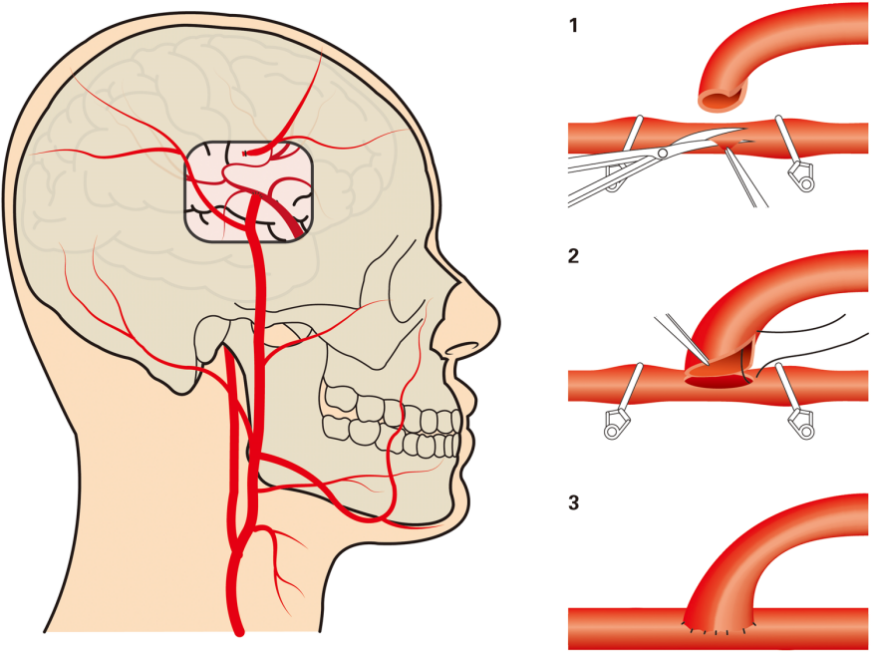
Schematic diagram of STA-IC bypass. STA-IC indicates superficial temporal artery to an intracranial artery.

Extracranial-to-intracranial (EC-IC bypass) was first reported by Crowell and Yasargil in 1969 for treating complex IAs.^36^ It isolates of the lesion via occlusion of the inflow artery and resumes regional circulation via a bypass from an extracranial artery to the distal branch of the occluded artery.^37^ There are 2 types EC-IC bypasses. The superficial temporal artery (STA) to an intracranial artery (STA-IC) bypass (Figure 4) is known as the low-flow bypass.^38^ The other is a high-flow bypass which connects the common carotid artery (CCA) or external carotid artery (ECA) to an intracranial artery (CCA-IC or ECA-IC) using a conduit: the great saphenous vein (GSV) or radial artery (RA).^39,40^ High-flow bypass is not preferable, as sudden increase in inflow rate usually causes hyperperfusion damage; moreover, a study by Sekhar et al revealed that inserting a high-flow bypass graft into MCA branches with a diameter less than 2 mm could cause significant local flow disturbances.^11^

The EC-IC bypass is essential in managing GIAs by resuming regional circulation distal to the lesion after eliminating the mass effect. This also makes it the only viable management for GSAs, as lesion removal is deemed necessary. A study showed EC-IC bypass as a treatment for GSA with a successful patency of 89.2% and a dramatically improved mortality rate to only 5.6%.^10^

There is also a patching technique for EC-IC bypass. The rationale is to graft a well-vascularized soft tissue which is still connected to the extracranial circulation into the designated brain region. After some time, peripheral circulation would be developed, bridging the extracranial and intracranial circulation.^41^ This is a much simpler technique than microvascular anastomosis. However, it takes time for the peripheral circulation to develop, which means the occlusion of aneurysmal artery or removal of the aneurysmal lesion would have to be a second stage of the 2-stage operations. Therefore, it is rarely used in managing IAs. Currently, it is only recommended for treating ischemic stroke, especially Moyamoya diseases.^42,43^

### Intracranial-to-Intracranial Bypass

In contrast to the EC-IC bypass, intracranial-to-intracranial (IC-IC) bypass is for in situ bypass. It consists of excision of the lesion and recanalization of the inflow and outflow arteries, with or without grafting.^44^ It requires the donor and recipient arteries to lie parallel and in close proximity to allow a tension-free anastomosis. There are 4 common sites in the cerebral circulation that are anatomically suitable for IC-IC bypass: (1) the ACAs, A2 and A3 segments, as they course over the genu and rostrum of the corpus callosum; (2) the MCA branches, through the sylvian fissure; (3) the posterior cerebral artery (PCA) and superior cerebellar artery, the section around midbrain through ambient cistem; and (4) the posterior ICAs (PICAs), around the posterior medulla and the tonsils in cisterna magna. In the cases where a tension-free anastomosis is not possible, GSV or RA grafts are used.^45^

Currently, the recommended treatment for BBAs is parent artery sacrifice with interpositional RA bypass graft.^46^ There has also been reports on GIAs and fusiform IAs being managed with IC-IC bypass^47^; however, it is not conventional due to the mismatch of the diameter of the 2 ends of arteries.

### Bipolar Coagulating for Microaneurysms

Microaneurysms do not require aggressive surgery. In cases where surgery is inevitable, such as recurrent SAH, clipping is not suitable for their miniature nature; instead, direct bipolar coagulating is highly effective.^48^ It is important to differentiate small BBAs from microaneurysms. While they resemble each other on imaging and other characteristics, direct bipolar coagulating of BBAs can lead to massive bleeding.

## Transluminal Embolization Techniques

### Simple Coiling

Detachable coils were invented by Guglielmi in the 1990s, and transluminal embolization techniques were gradually developed since then.^49^ Simple coiling refers to transluminal navigation of a microcatheter into the aneurysmal dome with the help of microguidewires and the delivery and packing of detachable coils within the aneurysmal sac. The goal in coiling is to achieve dense packing and induce rapid blood clot formation within the aneurysmal sac, hence isolating it from active circulation. Simple coiling is suitable for all IAs with desirable dome-to-neck ratios (>2.0), excluding BBAs as their fragile wall poses high risks of perforation.^50^

### Double Catheter Technique

Double catheter technique is for IAs with a slightly unfavorable dome-to-neck ratio (≤2.0, >1.5). Before coiling, the 2 microcatheters are positioned in the proximal and distal aspects of the aneurysmal dome. The first coil is deployed in proximal to create a supporting frame, and then the rest of the coils are deposited via the distal microcatheter. The framing coil is not detached until satisfactory packing is obtained. This technique is safe and effective for elongated IAs, especially for those at MCA bifurcations. However, there are some concerns on coils shifting at the withdrawal of the distal microcatheter.^51^

### Balloon-Assisted Coiling

**Figure 5.**
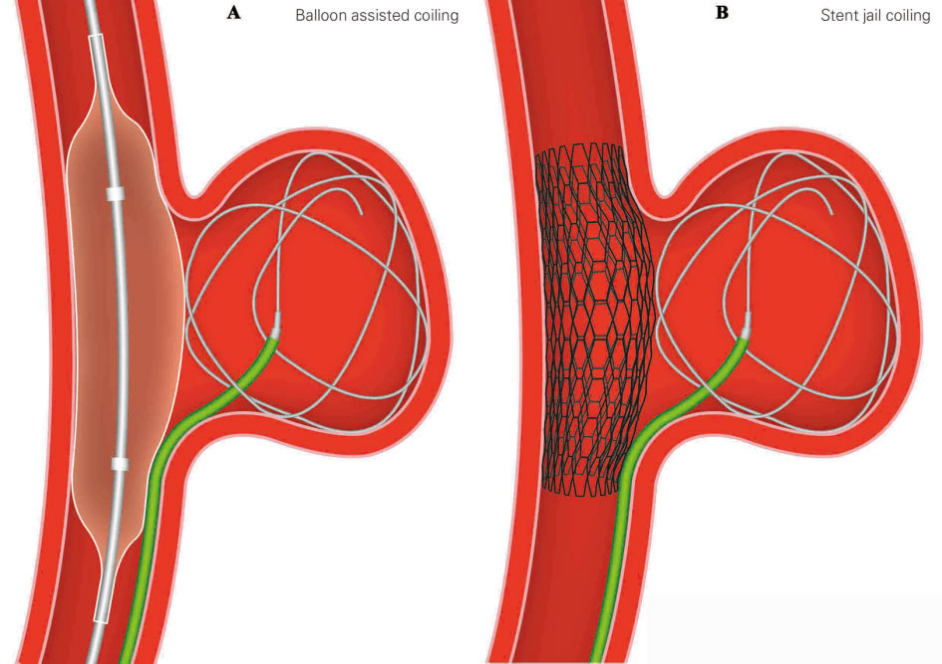
Schematic diagram of BAC (A) and stent jail techniques (B) for wide-necked IAs. BAC indicates balloon-assisted coiling; IAs intracranial aneurysms.

Balloon-assisted coiling (BAC) was initially described by Moret et al in 1997 in treating IAs with a wide neck.^52^ It is described as using 1 or multiple nondetachable temporarily inflated balloons to block the aneurysmal neck during coil placement (Figure 5A). For difficult situations or complex cases, multiple balloon technique is used. Besides multiple balloon technique, special balloons are also being developed, such as hypercompliant, round-shaped, and double lumen balloons.

The BAC was used frequently in IAs with unfavorable dome-to-neck ratio (≤1.5, >1.0). Analysis of Treatment by Endovascular approach of Non-ruptured Aneurysms (ATENA) revealed that intraoperative aneurysmal rupture rate was higher in BAC group than simple coiling (3.2% vs 2.2%) and BAC was associated with higher permanent morbidity and mortality; however, the study was underpowered to find the difference be significant.^53^ Clinical and Anatomical Results in the Treatment of Ruptured Intracranial Aneurysms (CLARITY) also suggested higher thromboembolic rate (12.7% vs 11.3%), morbidity (3.9% vs 2.5%), and mortality (1.3% vs 1.2%) in BAC group than simple coiling.^54^

### Stent-Assisted Coiling

The first report of stent-assisted coiling (SAC) for IAs was also in 1997, published by Higashida et al.^55^ The SAC can overcome the limitations of wide-necked, gigantic, fusiform, and some other complex IAs.^56^ Similar to BAC, a stent is deployed to block the aneurysmal neck before coil packing. The IAs with an extremely unfavorable dome-to-neck ratio (≤1.0) require SAC generally due to the need of permanent support to prevent coil prolapse and migration. There are 4 major SAC techniques.

### Simple SAC

This is also referred to as mesh technique. A stent is deployed as the first step and then a microcatheter is navigated into the aneurysmal lumen via the mesh of the stent. Coils are delivered through the microcatheter. However, it requires high level of guidewire navigating skill from operators, and it can be difficult to maintain the microcatheter’s position during coil deployment.^57^

#### Stent jail technique

Similar to mesh technique, the microcatheter is positioned before the stent bridging over the aneurysmal neck (Figure 5B). There is no difficulty in holding the microcatheter’s position during coil packing as it is trapped by the stent. However, stent migrations were reported to have occurred during retrieval of the microcatheter.^58^ This is of less concern nowadays because of retrievable stents such as the Enterprise (Cordis, Florida) and the Solitaire (ev3, Irvine, California). They contribute to semi-jailing technique where the stent is semideployed during coiling and only fully deployed after retrieval of the microcatheter. This enables readjustments of the stent position in case of migration. It is safe and effective, and it is one of the most used SAC techniques nowadays.^59^

#### Stent jack technique

**Figure 6.**
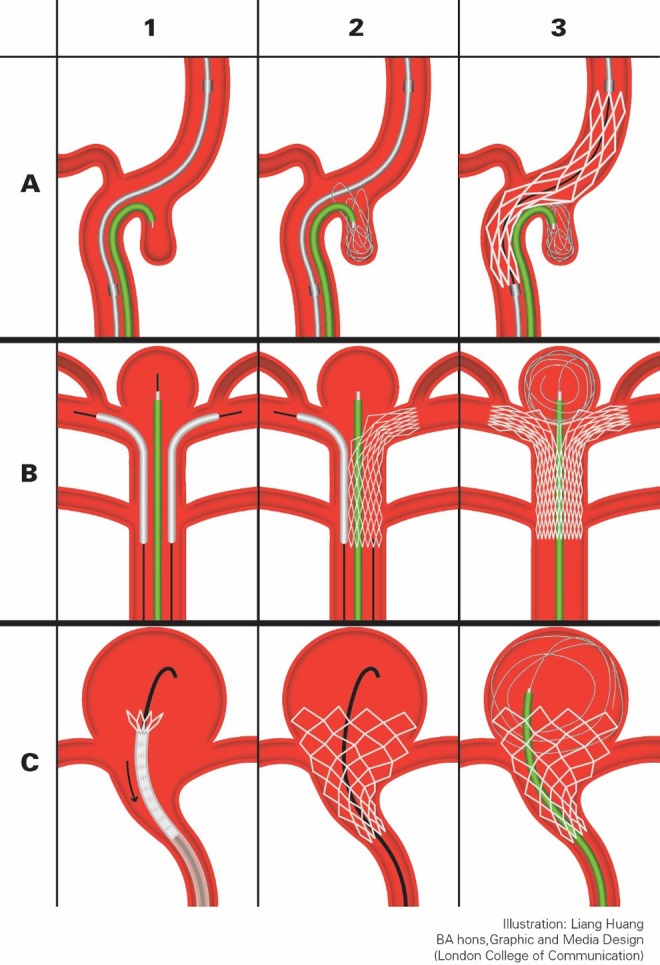
Schematic diagrams of special SAC techniques. A, stent jack: (A1) self-expandable stent navigated across the aneurysmal neck and microcatheter into the aneurysmal sac; (A2) first coil deployed; (A3) Stent deployed fully (or partially) bridging the neck, pushing the coil into the sac. B, Y-stenting: (B1) microcatheter navigated into the aneurysmal sac while 2 Neuroform stents navigated through the BA and respective PCA via exchange wires; (B2) 1 stent deployed; (B3) contralateral stent deployed in a “kissing” fashion. C, Waffle-cone technique: (C1) Enterprise stent and exchange-wire positioned; (C2) stent deployed; (C3) microcatheter positioned, coils deployed in waffle-cone configuration. SAC indicates stent-assisted coiling; BA, basilar artery; PCA; posterior cerebral artery.

In this technique, the stent delivery system and the microcatheter are both in place as the first step, and the stent is deployed after the deployment of the first coil in the aneurysmal sac.^60^ It allows the first coil to form a larger loop which is then pushed back into the sac, resulting in better coil wall positioning (Figure 6A). There is also a semi-jacking technique with retrievable stents, where the stent is partially deployed in the jacking movement. It allows multiple jacking. However, it is not recommended for the increased risks of rupture and the possibility of compromising the stent’s integrity and stability.^58^

#### Y-stenting technique

Y-stenting technique is developed for treating bifurcation IAs, where 1 or more microcatheter are in place with 2 stents blocking the aneurysmal neck (Figure 6B).^61^ It is by far the best technique for treating bifurcation basilar artery aneurysms. Their anatomic position makes it difficult to deal with via other techniques: They are usually in close relation with the root of the PCAs, it is impossible to block the neck with just 1 stent without leaving the contralateral PCA vulnerable to coil prolapsed and migration. Normally, nonretrievable open cell stents such as Neuroform (Boston Scientific Neurovascular, Fermont, California) or Wingspan (Striker/Boston Scientific SMART, Fermont, California) are used. Experienced operators would use slow motion to deploy the stent so that stent structures overlay closer to each other, creating a better wall effect.

#### Other SAC techniques

There are many other stenting techniques based on similar principles, one of those is the waffle-cone/ice-cone technique.^62^ It is described in case studies using the Neuroform or Enterprise stent, where the stent is deployed into the proximal neck of the aneurysm and the coils are packed in a waffle-cone conformation (Figure 6C). This technique is suitable for large wide-necked bifurcation IAs. It requires the flare ends of the stent (4.5 mm) be wider than the aneurysmal neck. Compared to the Y-stenting, this approach uses a single stent and thus reduces risks of thromboembolic events and the probability of in-stent stenosis. It is more flexible and can be used in a wide range of vessel configuration, but it is also technically demanding on operators. It is currently recommended for IAs with elongated domes, wide necks, and on bifurcating vessels where Y-stenting or surgical clipping are unsuitable.^62^ In IAs with shorter dome heights, the forward tension on the microcatheter could result in the backward migration of the stent during coiling.

### Flow-Diverting Stent

Flow-diverting stents (FDSs) are a new generation of stents designed to treat IAs by isolating the aneurysmal lumen from the circulation via recanalization.^63^ They are either braided mesh stents, such as the Silk Flow Diverter (Balt Extrusion, Montmorency, France), the Pipeline Embolisation Device (ev3, Irvine, California), or covered stents such ad the Willis Covered Stent (MicroPort Medical Company, Shanghai, China).^64^

**Figure 7.**
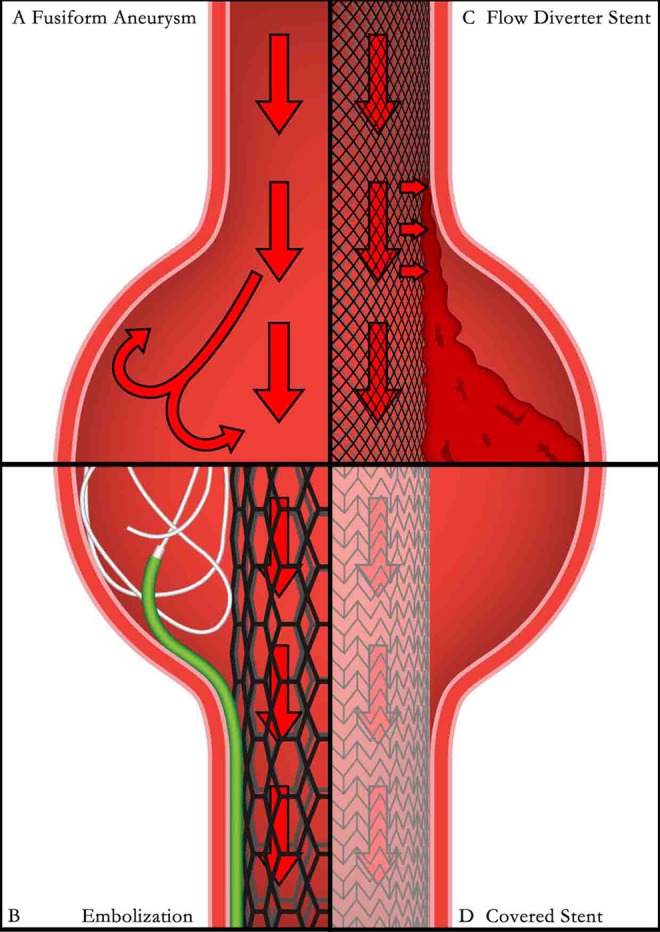
Schematic diagram of treatment strategies for fusiform IAs. (A) hemodynamics within a fusiform IA; (B) SAC for fusiform IA; (C) braided mesh stent for fusiform IA; and (D) covered stent for fusiform IA. IAs indicates intracranial aneurysms; SAC, stent-assisted coiling.

The FDSs are suitable for both wide-necked and fusiform IAs. In fusiform IAs, turbulence is formed due to their unique geometric shape (Figure 7A), conventional SAC requires dense packing of coils in a hula-like domain (Figure 7B). It is difficult to be achieved even by skilled operators, and usually demand both multiple stents assisting and multiple microcatheters for coil delivering to cover insidious or edging spaces. The main concern with FDSs is the risk of perforator blockage which makes them less desirable in treating general wide-neck IAs. However, in fusiform IAs, FDSs are advantageous as the perforators located in the lesion are considered compromised (Figure 7B shows a fusiform IA being treated by a braided mesh stent, and Figure 7D shows it being treated by a covered stent). Few reports mentioned selective clipping being applied to preserve important perforators in fusiform IAs; however, the outcomes were unsatisfactory as residual diseased segments were preserved and subsequently caused saccular IA formation in situ or fusiform IA formation of the preserved perforators.^65^

The FDS is also suggested for BBAs. Multistent reconstruction and stent-in-stent technique were reported for treating BBAs before FDS.^66^ Despite the lack of data of BBAs treated with FDSs from large series clinical studies, a few recent small clinical studies or case reports suggested FDS to be a safe and feasible alternative for BBAs.^67–70^ Especially, when comparing to other surgical approaches, as pointed out by Aydin et al in 2015, BBAs clipping was a predictor of intraoperative bleeding (odds ratio [OR] 6.5; 95% confidence interval [CI] 1.2-34.3), while SAC increased the likelihood of a second treatment (OR 4.1; 95% CI 1.3-13.1), its conversion to another modality (OR 4.7; 95% CI 1.4-16.0) and incomplete aneurysm obliteration (OR 2.6; 95% CI 1.0-6.6).^71^ However, the use of antiplatelet therapy after FDS treatment for BBAs still remained controversial.^67^

### Simple Stenting for Intracranial Dissecting Aneurysms

For intracranial dissecting aneurysms, simple stenting is often used as the most effective approach to trap the flap and close the tear, restoring wall integrity. However, it is essential to identify the true lumen from the pseudolumen before stenting, which could be challenging under circumstances.^72^ Open surgery is seldom used to treat intracranial dissecting aneurysms; however, Wu and Chin in 2013 reported a rare case of a large dissecting aneurysm in A1 segment of ACA, where surgical option was selected in order to treat the aneurysm and to eliminate the mass effect.^73^

### Salvation Techniques

Salvation techniques are for dealing with ruptured IAs or vascular trauma during interventional procedures.^57^ The principle of salvation technique is to restore the integrity of vessel walls and stop the bleeding. The most commonly used one is to deploy a covered stent to block the perforation, sacrificing in situ perforators. Overlapping stenting and FDSs are also used to close the defect by trapping tissues with dense stent structure, and it is safer for preserving perforators. In extreme scenarios, a detachable balloon is used to occlude the entire parent artery as a life-saving procedure to stop bleeding. This would cause severe ischemic complications and therefore is only used as a last resort.

### Intrasaccular Flow Disruptions

Besides detachable coils, other types of intrasaccular flow disruption devices are also being developed, among which is the Woven EndoBridge Embolization Device (Sequent Medical, Aliso Viejo, California). It is to be deployed inside the aneurysmal sac to induce fast thrombosis.^74^ It is suitable for most saccular IAs and even ruptured IAs, as it facilitates acute aneurysmal occlusion. It does not place adjacent perforating arteries at risk, and there is no need for antiplatelet therapy following the procedure.^75^ Two single-center series demonstrated high-technical success of treatment with no mortality and morbidity less than 5%.^76^ However, some experts point out its limitation in treating irregular dome-shape IAs as good wall apposition is impossible.

### Liquid Embolic Material

A bold attempt was made to use liquid embolic agent as intrasaccular filling. Onyx (Covidien/EV3, Irvine, California) is a liquid embolic filler containing ethylene vinyl alcohol (EVOH) copolymer and dimethyl sulfoxide (DMSO) in a volume ratio of 3:2 and tantalum powder (28%, wt/wt) as radiopaque marker.^77^ Once contacted with iron-containing bodily fluids, DMSO will dissipate while EVOH and tentalum will form a spongy solid filling.^78^ During the procedure, a remodeling balloon is used to block the aneurysmal neck while Onyx is progressively injected into the sac. It is extremely suitable for complex irregularly shaped IAs. However, concerns arise where fragments of filling may break off and become emboli after withdrawal of balloon.^79^ Currently, Onyx is recommended in the embolization for intracranial arteriovenous malformation.

## Other Treatment Strategies

### Regular Follow-Ups

Regular angiogram follow-ups are proposed and supported by more and more clinicians as screenings for de novo aneurysms. The American Stroke Association (ASA) guidelines recommend a 6- to 12-month interval for angiographic follow-up such as Computer Tompgraphy Angiography (CTA), Magnetic Resonance Angiography (MRA) or DSA for at least 5 years following effective aneurysmal treatment.^80,81^

### Wait and See Strategy for Microaneurysms

Controversy exists as to whether or not microaneurysms should be treated aggressively. Although the rupture rate of microaneurysms is undetermined due to lack of data from large clinical studies, data from case reports suggest microaneurysms with a diameter of less than 1 mm have rupture rate close to 0.^82^ For this reason, some specialists suggest a wait and see strategy instead of immediate intervention. However, opposite opinions are also held for being exposed to the shearing force from circulation, microaneurysms may grow in time into saccular IAs and thus should recommend early treatment.^80^

### Targeting Therapy for IIAs

The principle for treating IIAs is managing primary infection for open surgery is to be avoided due to its infectious nature. Many case reports have described unruptured IIAs decreasing in size following administration of appropriate antibiotics.^83^ There have been a few successful reports in recent years on proximal clip occlusions with or without bypass for ruptured IIAs; however, these microsurgeries are challenging in general.^84^

## Treatment-Related Complications

### Delayed Cerebral Ischemia

Delayed cerebral ischemia (DCI) is a serious complication arising from SAH, and its pathophysiology is unclear. Previous studies suggest multiple factors contribute to DCI, including vasospasm and microvascular thromboembolism.^85^ It is more common in patients treated with surgical clipping than transluminal approaches.^86^

### Hydrocephalus

Hydrocephalus is one of the major complications of SAH. There are 2 types of hydrocephalus following SAH, acute and chronic. Acute hydrocephalus usually occurs before day 7 of initial SAH; it is caused by the obstruction of cerebrospinal fluid (CSF) flow by blood degradation products when the ventricle is breach. External ventricular shunt is routinely performed for Fisher grades III and IV SAH to prevent acute hydrocephalus, followed by clamp test on day 15 before it is possibly removed. Chronic hydrocephalus tends to occur after day 30 and is due to disturbance of normal CSF reabsorption from the arachnoid space. Depending on age and clinical signs, the treatment of chronic hydrocephalus is either a depletional lumbar puncture or a ventricular peritoneal shunt.^87^ In a cross series of 7 studies (total 1981 patients), the rates of developing chronic hydrocephalus after clipping or coiling are16.4% and 19.3%, respectively.^86,88^

### Vasospasm

Vasospasm is usually triggered by intensive stimuli on vessel walls during clipping and the manipulation of endovascular devices. It could cause diffuse microvascular thromboembolism in severe cases.^89^ Postoperative transcranial Doppler monitoring is becoming a routine as an effective monitoring and detection of severe vasospasm. A meta-analysis by Li et al in 2013 suggested the risk of vasospasm is higher in ruptured IAs treated by surgical clipping than coiling.^86^

### Seizures

Seizures are a common complication of craniotomy as a result of disruption to the cerebral cortex.^90^ In the past, transluminal treatments of unruptured IAs were disassociated with postoperative seizures.^91^ However, recent studies suggest such risks do exist, with rates varied substantially among published reports from 0.01% to 6.2%. Lai et al in 2013 compared the risk of postoperative seizures in unruptured IAs, the results of which suggested that elective surgical clipping has a higher risk of postoperative seizure than transluminal coiling.^92^

### Stent-Related Complications

Despite the generations of intracranial stents which have evolved over time, there are still a number of limitations with the current stent devices, including stent displacement and migration, vessel trauma, thrombosis, and in-stent restenosis. Thromboemboli rate after stent placement is around 10% with current commercially available intracranial stents, the documented in-stent stenosis rate is around 8%, while morbidity and mortality rates are 5% and 3%, respectively.^64^

### Coil-Related Complications

Coil migration is one of the most concerning complications with embolization. Coil migration rate was 2.3% in the initial report by Guglielmi et al.^93^ It could cause infarcts with asymptomatic strokes in minor cases, while in extreme cases, it could occlude major branches resulting in large territory infarcts. Majority of coil migrations occur during embolization procedure. Delayed coil migrations are considered to be hemodynamic related. Henkes et al in 2006 were first to report retrieving migrated coils with the Alligator Retrieval Device (Chestnut Medical Technologies, Menlo Park, California), a device comprised of precision grasping arms tailed by a 0.016-in stainless steel insertion wire.^94^ Later on, successful coil retrievals with Merci Retriever (Concentric Medical, Mountain View, California), Solitaire stent (ev3, Irvine, California), Catch Plus (Balt Extrusion, Montmorency, France), and so on were reported by different authors.^95^

### Delayed Aneurysm Rupture

Delayed aneurysm rupture (DAR) is a complication associated with IAs treated with FDSs. A study by Rouchaud et al. in 2016 reviewed 35 individual studies of IAs treated with FDSs and found a total of 81 documented cases of DAR. Among these, the incidence of delay in rupturing for less than 1 day, between 1 day and 7 days, from 7 days to 1 month, and more than 1 month were 6 cases (10.3%), 19 (32.8%), 20 (34.5%), and 13 (22.4%), respectively. However, the overall incidence of DAR in IAs treated with FDSs was not included in the study.^96^ Although the mechanism of DAR is still poorly understood, Shobayashi et al in 2013 pointed out that after FDS treatment, intra-aneurysmal pressure did not decrease although the volume and velocity of blood flowing into the aneurysmal lumen were reduced; this might be the pathophysiology of DAR.^97^

## Discussion

### Clipping or Coiling?

The debate regarding the safest and most effective therapeutic strategy in patients with IAs is ongoing. The Internal Subarachnoid Trial (ISAT) completed in 2002 showed that transluminal coiling had a better treatment outcome than surgical clipping. However, it received heavy criticisms for recruitment bias, as only 2143 out of 9559 screened patients were included.^98^ In responding to that, the CLARITY was initiated in France and Barrow Ruptured Aneurysm Trial (BRAT) in Phoenix, Arizona. They both confirmed ISAT conclusion; despite a high rate of treatment crossover, patient morbidity and mortality at 1-year follow-up were better after coil embolization than surgical clipping.^99^ Currently, ASA guidelines and National Institute for Clinical Excellence (NICE) guidelines recognize both surgical clipping and endovascular coiling as effective treatments for ruptured and unruptured IAs (class I; level of evidence B), and a “coil first” strategy is recommended for both ruptured and unruptured IAs. The guidelines and their supporting evidence emphasize the findings in major clinical study series reported between 1990 and 2001: Coiling shows superiority over clipping in both morbidity and mortality for unruptured IAs, while coiling shows no less favorable outcome compared to clipping for ruptured IAs.^80,81,100,101^

Chen et al in 2012 summarized and compared the results from 4 single-center studies on treating anterior circulation IAs with surgical clipping and transluminal approaches and found no significant difference in patient outcomes in terms of morbidity and mortality in follow-ups.^102^ However, a meta-analysis by Li et al in 2013 found that aneurysmal recurrence in patients following transluminal embolization was significantly higher than surgical clipping.^86^ These findings suggest surgical clipping may be superior in treating anterior circulation IAs with low recurrent rates, while posterior circulation lesions may be better accessed and treated endovascularly.

In 2013, Lad et al compared the long-term economic impact of coiling versus clipping of unruptured IAs. Patients who underwent coiling were more likely to undergo a secondary operation than those who underwent clipping surgery at 1 year (odds ratio: 2.73, 95% confidence interval [CI] 1.86-4.00, *P* < .001) and more likely to undergo postoperative angiograms (OR: 3.73, 95% CI 2.87-4.86, *P* < .001). Patients who underwent surgical clipping had a significantly longer hospital stay compared to coiling (5 vs 2 days, *P* < .001), resulting in higher initial hospital costs ($41 386 vs $36 101, *P* < .001). The overall costs of clipping at 2 and 5 years were similar to coiling due to high number of follow-up angiograms and outpatient costs ($79 577 vs $82 986, *P* = .69).^103^

## Conclusion

**Figure 8.**
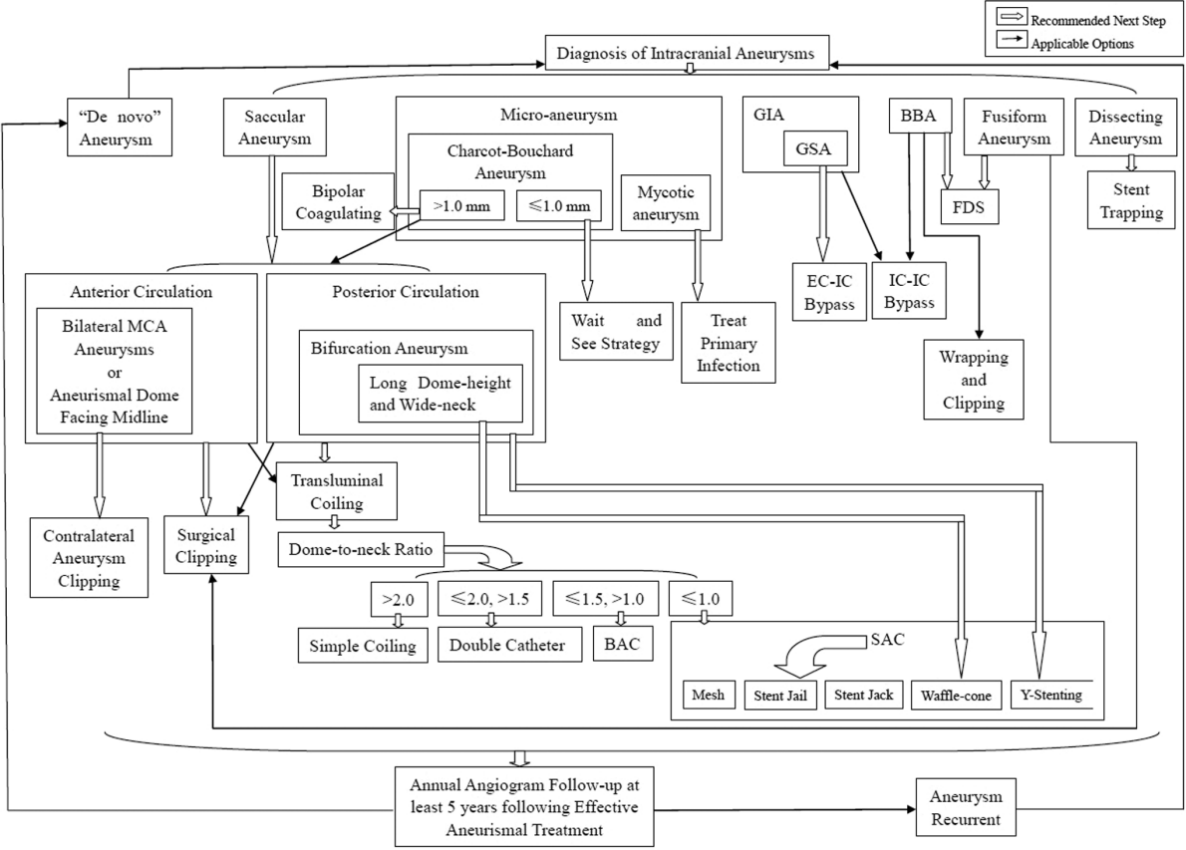
A suggested clinical pathway for unruptured IAs. IAs indicates intracranial aneurysms.

The preferred treatment strategy of unruptured IAs remains uncertain. Management decisions have to take into consideration morphology of the aneurysm and patients' co-morbidities and preferences, taking within a multidisciplinary team. Based on findings from recent clinical studies, a clinical pathway for the management of unruptured IAs has been proposed, as shown in Figure 8. Surgical clipping may be preferable for anterior circulation IAs based on findings of comparable clinical outcome, low recurrence rate and cost effectiveness, whilst endovascular coiling for posterior circulation IAs is less invasive and associated with better clinical outcomes. For more complex cases, different approaches should be considered, including lesion removal together with surgical bypass for GIAs and GSAs, and FDSs for fusiform IAs and BBAs. As endovascular techniques and devices continue to evolve, management options of unruptured IAs are likely to expand with improved outcomes.

For ruptured IAs with SAH, both ASA and NICE guidelines suggest that endovascular coiling should be considered instead of surgical clipping (class I; level of evidence B). Stenting is also associated with increased morbidity and mortality in ruptured IAs; therefore it should be avoided (class III; level of evidence C). The patient’s condition at presentation plays an important role in the decision making by experienced neurovascular surgeons and endovascular specialists.
